# A feedback framework for protein inference with peptides identified from tandem mass spectra

**DOI:** 10.1186/1477-5956-10-68

**Published:** 2012-11-19

**Authors:** Jinhong Shi, Fang-Xiang Wu

**Affiliations:** 1 Division of Biomedical Engineering, University of Saskatchewan, 57 Campus Dr, Saskatoon, Canada; 2 Department of Mechanical Engineering, , 57 Campus Dr, Saskatoon, Canada

## Abstract

**Background:**

Protein inference is an important computational step in proteomics. There exists a natural nest relationship between protein inference and peptide identification, but these two steps are usually performed separately in existing methods. We believe that both peptide identification and protein inference can be improved by exploring such nest relationship.

**Results:**

In this study, a feedback framework is proposed to process peptide identification reports from search engines, and an iterative method is implemented to exemplify the processing of Sequest peptide identification reports according to the framework. The iterative method is verified on two datasets with known validity of proteins and peptides, and compared with ProteinProphet and PeptideProphet. The results have shown that not only can the iterative method infer more true positive and less false positive proteins than ProteinProphet, but also identify more true positive and less false positive peptides than PeptideProphet.

**Conclusions:**

The proposed iterative method implemented according to the feedback framework can unify and improve the results of peptide identification and protein inference.

## Background

Protein inference by assembling peptides identified from tandem mass spectra (MS/MS) is an important computational step in proteomics, based on which further analysis, such as inference of protein structure and function can be performed. Comprehensive discussion about this problem can be referred to [1-3]. Existing MS/MS-based methods to address this problem can be categorized into two groups. The first group performs protein inference and peptide identification separately [4-8]. Peptides are first identified from tandem mass spectra by de novo sequencing [9-11] or database search [12-14], and then proteins are inferred by assembling these identified peptides. The other group combines protein inference with peptide identification, identifying peptides and proteins simultaneously [15-17]. A Barista model [16] has been built to formulate the protein inference as an optimization problem. A tripartite graph is used to represent the protein inference problem, with layers corresponding to spectra, peptides and proteins. The input to Barista is the tripartite graph with a set of features describing the peptide-spectrum-match (PSM). The score of a PSM is computed with a nonlinear function based on the feature set, and the score of a peptide is the maximum PSM score of all spectra mapped to this peptide, then the score of a protein is the normalized sum of its constituent peptide scores. It is advantageous for this model to utilize the spectrum information in all the steps of its protein inference, without discarding spectra from peptide identification to protein inference. The parameters in the model are estimated by training the model with reference data, and then the trained model is used to infer proteins. Its application is limited by the requirement of reference data to train the model each time when a different dataset is analyzed.

Since many well-developed search engines for peptide identification are available, methods for processing the peptide identification reports from these engines have been proposed. As an example, a nested mixture model [17] has been used by Li *etc* to estimate peptide and protein probability simultaneously with identified peptides and their scores from search engines. This model allows evidence feedback between proteins and their constituent peptides. Several reasonable assumptions are adopted to build this model, except that the problem of shared peptides is completely ignored.

This paper proposes a unified framework to process peptide identification results from database search engines. The goal is to output a list of proteins and a list of corresponding peptides at the same time, and it is achieved by iteratively updating the two lists with a feedback from the inferred proteins to the selection of correct peptides. Specifically, the inferred protein sequences are used to search low-confidence peptides from the search engine and the probabilities of these peptides are recomputed. Different methods can be designed according to this framework for protein inference. Here, an iterative method is exemplified to process Sequest peptide identification reports based on the proposed framework. In addition, to address the challenge of assigning shared peptides, an MS/MS intensity-based strategy is proposed to compute the probabilities of shared peptides based on the closeness between the intensity of a shared peptide and the intensity of its siblings in parent proteins. We evaluate the iterative method on two datasets with known validity. The results have shown that not only can it infer more true positive and less false positive proteins than ProteinProphet [4], but also identify more true positive and less false positive peptides than PeptideProphet [18].

## Materials and methods

### Unified framework for MS-based protein inference

**Figure 1:**
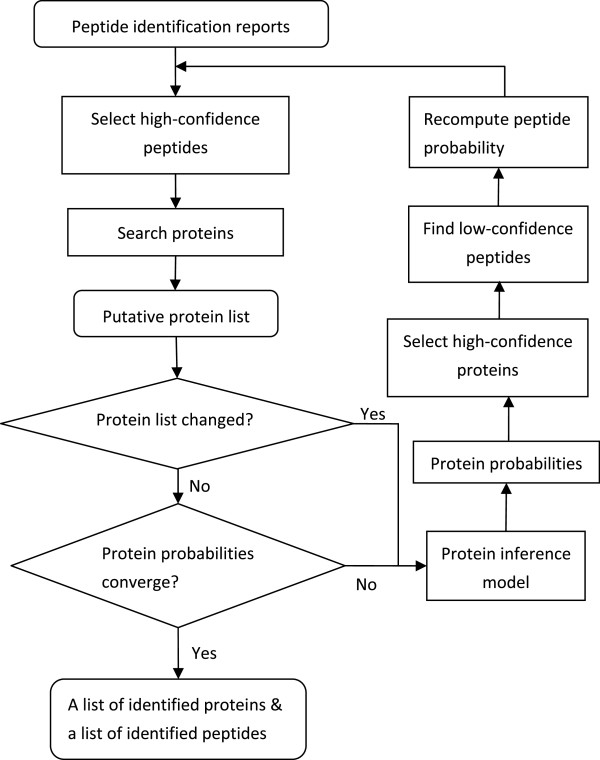
**Unified framework for MS-based protein inference.** This framework for protein inference starts with the peptide identification reports from search engines. It unifies peptide identification and protein inference by adding a feedback from identified proteins to the identification of peptides. The end products from the framework are a list of identified peptides and a list of counterpart proteins.

A unified framework for protein inference by assembling peptides identified from tandem mass spectra is introduced. Peptide identification and protein inference are combined together because there exists a natural nest relationship between these two computational steps in proteomics. The diagram of the framework is given in Figure 1. Here, the starting point is the peptide identification reports from search engines, such as Sequest or Mascot. The main operations in the framework include: (1) select high-confidence peptides to search proteins and produce a list of putative proteins; (2) compute protein probabilities; (3) use proteins with high-confidence to replenish the peptide list with previous low-confidence peptides, and recompute the probabilities of all selected peptides. These steps are repeated until the stop condition is reached. The feedback from protein inference to peptide identification can help to improve peptide identification results, and thus improve protein inference results as well. The computation will stop when protein probabilities converge, and then we can obtain the inferred proteins and identified peptides simultaneously.

In the following sections, an iterative method is implemented to process Sequest peptide identification reports according to the unified framework. A list of peptides and a list of proteins will be output simultaneously. The computation steps in the iteration process are introduced.

### Protein inference model

Given Sequest peptide identification reports, and the probabilities of peptide identifications computed with PeptideProphet [18], the protein inference model in the *n*^th^iteration is written as

(1) $$\begin{array}{ll} q_{k}^{\left(n\right)} & ={\text{Pr}(\text{Q}}_{k}={1|X}_{k}^{\left(n,-,1\right)},\frac{n_{k}^{\left(n,-,1\right)}}{N_{k}})\\ & =\;[1-\overset{n_{k}^{\left(n,-,1\right)}}{\underset{i=1}{\prod }}(1-\text{Pr}({\text{Peptide}\;\text{P}}_{i}\;{\text{from}\;\text{Q}}_{k}\;\\ & \;\;\;{\text{is}\;\text{correctly}\;\text{identified}))]}^{-\log\left({\text{n}}_{k}^{\left(n,-,1\right)}/N_{k}\right)}\\ & =\;[1-\overset{n_{k}^{\left(n,-,1\right)}}{\underset{i=1}{\prod }}(1-{\text{Pr}(\text{P}}_{i}^{\left(n,-,1\right)}=1,{\text{P}}_{i}^{\left(n,-,1\right)}\in {\text{Q}}_{k}^{\left(n,-,1\right)},\\ & \;\;\;{\text{Q}}_{k}^{\left(n,-,1\right)}={1))]}^{-\log\left({\text{n}}_{k}^{\left(n,-,1\right)}/N_{k}\right)}\\ & =\;{\left[1-\overset{n_{k}^{\left(n,-,1\right)}}{\underset{i=1}{\prod }}(1-x_{i}^{\left(n,-,1\right)}w_{i}^{k^{\left(n,-,1\right)}}q_{k}^{\left(n,-,1\right)})\right]}^{-\log\left({\text{n}}_{k}^{\left(n-1\right)}/N_{k}\right)}, \end{array}$$

where the superscripts (*n*) and (*n*−1) denote the index of iteration, and *n*≥1. In the following, for simplicity, we will only introduce the variables if it is not necessary to mention the index of iteration. *q*_*k*_ is the probability of protein Q_*k*_ being present in the sample; *n*_*k*_and *N*_*k*_are the number of experimental and theoretical peptides from protein Q_*k*_; *x*_*i*_ is the probability of peptide P_*i*_ being correctly identified, and $w_{i}^{k}$ is the probability that peptide P_*i*_comes from protein Q_*k*_, the computation of which will be introduced in the next section.

Here, it is assumed that the event “peptide P_*i*_is correctly identified, i.e. P_*i*_=1”, the event “peptide P_*i*_comes from protein Q_*k*_, i.e., P_*i*_∈Q_*k*_”, and the event “protein Q_*k*_ exists in the sample, i.e., Q_*k*_=1” are independent of each other, because whether peptide P_*i*_ is identified is not dependent on whether protein Q_*k*_is present in the sample. Peptide P_*i*_could be generated by other proteins. In addition, the number of theoretical peptides *N*_*k*_is included to factor the length of a protein in the model. It is computed based on these criteria: (1) trypsin-cutting; (2) two missed cleavages are allowed; and (3) peptides with masses falling in *M*_*min*_*M*_*max*_. The minimum *M*_*min*_ and maximum *M*_*max*_peptide mass are determined from the peptide identification reports. An alternative way is to only consider peptides with a certain length [16].

### The computation of $w_{i}^{k}$

It is difficult to compute the probabilities of a shared peptide belonging to different parent proteins, because the connection between peptides and proteins is lost in proteome experiments. Here we propose an MS/MS intensity-based strategy to assign shared peptides to truly present proteins. The idea is that, for a given peptide which is shared by protein Q_1_ and Q_2_, if the peptide was from Q_1_, then its intensity will be closer to the intensity of its siblings in Q_1_than that in Q_2_. Two peptides are siblings when they are from the same parent protein. The intensity of a peptide is computed with the signal peak intensity in its matched tandem mass spectra.

This MS/MS intensity-based method requires that all peptides in the sample have a similar ability to be ionized and fragmented, and thus have a similar chance to be analyzed by mass spectrometers. However, this is not the case in practice. One way to alleviate the effect of peptide detectability [19] on peptide intensity is that, for each protein with shared peptides, we compute the average intensity of peptide siblings and compare this intensity to the intensity of a shared peptide. Some peptides of a protein may have low detectability, but others may not. Thus, averaging the intensity of all peptide siblings can help to reduce the effect of detectability on intensity. An alternative way is to combine peptide detectability into the computation of peptide intensity, if the computation of detectability is accurate enough. Here, we use the first simple way and leave the second method to future investigate. The intensity of a peptide is computed as the sum of the signal peak intensity in all its matched tandem mass spectra, which is given by

(2) $$I_{p}=\overset{N_{s}}{\underset{i=1}{\sum }}S_{p_{i}},$$

where *I*_*p*_ is the peptide intensity and *N*_*s*_is the number of tandem mass spectra matched to the peptide. $S_{p_{i}}$ is the preliminary score in Sequest [13] output for the *i*^th^tandem mass spectrum, which is the sum of the intensity of all signal peaks in the spectrum. And it is factored with the ratio between experimental and theoretical peaks which can be derived from the peptide. This factor can eliminate the unfair advantage of longer peptides over short ones. In addition, we normalize $S_{p_{i}}$ with the maximum value in each whole data set.

As previously mentioned, for a given shared peptide, the intensity of its siblings is averaged in order to reduce the effect of peptide detectability on intensity. So the intensity of a shared peptide’s siblings is calculated by

(3) $$I_{b}=\frac{1}{N_{b}}\overset{N_{b}}{\underset{i=1}{\sum }}I_{p_{i}},$$

where *I*_*b*_ is the average intensity of a given shared peptide’s siblings, and *N*_*b*_is the number of its siblings. $I_{p_{i}}$ is the intensity of its *i*^*th*^peptide sibling.

**Figure 2:**
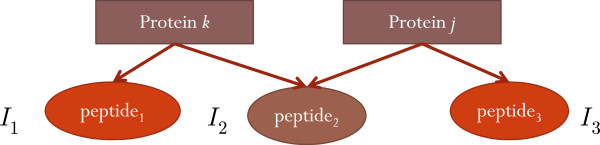
**An example of the assignment of shared peptides.** This example illustrates the computation of the probabilities that a shared peptide belonging to its parent proteins. *I*_1_, *I*_2_, and *I*_3_are the respective intensity of the three peptides.

The intensity of a shared peptide is contributed by all of its parent proteins in the sample. This makes the intensity proportion contributed by each protein sum to unity. A simple example is used to illustrate how to compute these proportions. In Figure 2, peptide P_2_ is shared by protein Q_*k*_ and Q_*j*_. The proportion contributed by protein Q_*k*_ to the intensity of peptide P_2_is calculated by

(4) $$P_{2}^{\mathrm{k\prime}}=\frac{\left|I_{2}-I_{3}\right|}{I_{2}},$$

where |·| is the absolute value operator. Similarly, the proportion contributed by protein Q_*j*_ is given by

(5) $$P_{2}^{\mathrm{j\prime}}=\frac{\left|I_{2}-I_{1}\right|}{I_{2}}.$$

Since the proportions contributed by all proteins sum to 1, the previous proportions are normalized,

(6) $$P_{2}^{k}=\frac{P_{2}^{\mathrm{k\prime}}}{P_{2}^{\mathrm{k\prime}}+P_{2}^{\mathrm{j\prime}}},\;\;\;P_{2}^{j}=\frac{P_{2}^{\mathrm{j\prime}}}{P_{2}^{\mathrm{k\prime}}+P_{2}^{\mathrm{j\prime}}}.$$

Here, we take these proportions to represent the probabilities of P_2_ belonging to protein Q_*k*_ and Q_*j*_, respectively. The probability for any peptide P_*i*_, unique or shared, belonging to any protein Q_*k*_is given as

(7) $$w_{i}^{k}=\left\{\begin{array}{l} P_{i}^{k}\;\;{\text{P}}_{i}\;\text{is shared by}\;{\text{Q}}_{k}\\ 1\;{\text{P}}_{i}\;\text{is unique to}\;{\text{Q}}_{k}. \end{array}\right)$$

During the iteration, after computing the probabilities of proteins, high-confidence proteins are selected to replenish the list of peptides, and new group of proteins and peptides are used to update the values of $w_{i}^{k}$ and *n*_*k*_.

It is worth pointing out that although the probabilities of shared peptides also sum to 1 as in ProteinProphet [4], it is not required that these shared peptides can only come from one truly present protein in the sample. In the case of ProteinProphet, the weights of a shared peptide will eventually be one of them is or close to 1, and the others are or close to 0, because it assumes that shared peptides can only come from one truly present protein. This is not true in practical experiments and also misinterprets the real meaning of shared peptides. Based on this assumption, a shared peptide can only come from one truly present protein in the sample; it is shared because it can be also generated by some other proteins in the chosen database. By removing this assumption, the probability $w_{i}^{k}$ allows shared peptides to be assigned to multiple proteins in the sample, as long as these proteins have enough evidence to support their existence.

### Recompute peptide probability

After we obtain the probability of all proteins in the *n*^th^ iteration, for each peptide P_*i*_, we find all of its parent proteins, and recompute its probability *x*_*i*_as follows

(8) $$x_{i}^{\left(n\right)}=\text{Pr}({\text{P}}_{i}=1|Q_{i1},{\text{Q}}_{i2}\ldots {\text{Q}}_{iM_{i}})=\overset{M_{i}}{\underset{k=1}{\sum }}w_{i}^{k^{\left(n\right)}}q_{k}^{\left(n\right)},$$

where the superscript (*n*) is the index of iteration; *M*_*i*_is the number of inferred parent proteins of peptide P_*i*_; $w_{i}^{k}$ and *q*_*k*_ are defined in Equation(7) and Equation(1), respectively.

To this point, we have introduced all the computational steps in the iteration process. The initial protein probabilities are set to the same of the value 1 by assuming that each protein has the same chance to be present in the sample as long as it has constituent peptides being identified. The initial values of peptide probability *x*_*i*_is the probability output by PeptideProphet, and the initial values of $w_{i}^{k}$ and *n*_*k*_ are computed from the Sequest reports.

### Experimental data

Two datasets are analyzed with the proposed method, and they were described in [20]. These datasets are adopted because they are collected specifically for verifying algorithms of protein inference and peptide identification. The search results are also provided along with these datasets, which makes it easier to be used as reference data. Database search for peptide identification was done with Sequest [13], and the statistical analysis of identification results was done with PeptideProphet [18]. Notice that some possible contaminants are considered in the datasets [20], and the summary of the two datasets is given in Table 1. 

**Table 1:** Statistics of ISB standard protein mix datasets

	**MS/MS**	**Standard Proteins**	**Contaminants**	**T&U**^**1**^	**F&U**^**2**^	**T&S**^**3**^	**F&S**^**4**^
Mix1	86850	18	13	1168	990	50	9
Mix2	83293	18	15	1773	2060	73	21

This table gives some statistics of the ISB standard protein mix datasets, including the number of tandem mass spectra, the number of standard proteins, the number of contaminant proteins and the number of peptides.

^1^True and Unique peptides.

^2^False and unique peptides.

^3^True and Shared peptides.

^4^False and Shared peptides.

### Evaluation of the method

The proposed method is compared with PeptideProphet [18] and ProteinProphet [4] for the peptide identification and protein inference, respectively. Specifically, we compare the number of true positive and false positive peptides and proteins produced from these methods.

## Results and discussion

### Protein inference results

**Figure 3:**
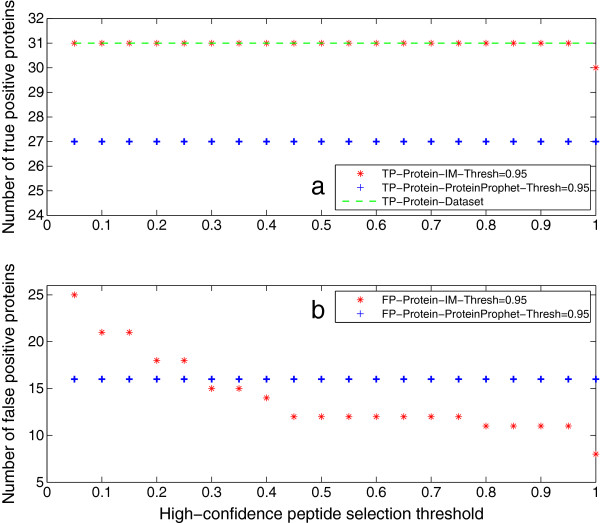
**Protein inference results of Mix 1.** This figure shows the protein inference results of Mix 1. The protein identification threshold is set as 0*.*95. Figure 3**(a)** gives the number of true positive proteins identified by the iterative method (IM) and ProteinProphet. It can be seen that IM always identifies more true proteins than ProteinProphet. When the high-confidence peptide selection threshold is less than 1, all the 31 true proteins can be identified; while 30 proteins can be identified when the threshold is 1. This indicates that all peptides identified for the missed protein have probabilities less than 1. Meanwhile, ProteinProphet can only identify 27 of the 31 true proteins. Figure 3**(b)** shows that the number of false positive proteins output by IM decreases with the increase of high-confidence peptide selection threshold, however, ProteinProphet outputs the same number of false positive proteins regardless of the threshold. The reason is that higher selection thresholds reject more false peptides to be included in the iteration process, and thus less false proteins will be identified by IM. On the other hand, some false positive proteins are removed with the elimination-rule in the iteration process, while they are retained by ProteinProphet.

The following sections will demonstrate the processing results of Sequest peptide identification reports with the proposed iterative method. First, we show the protein inference results. Figure 3(a) shows that the iterative method always identifies more truly present proteins in Mix 1 than ProteinProphet. When the threshold for selecting high-confidence peptides is less than 1, all the 31 true proteins can be identified by the iterative method; while 30 proteins can be identified when the threshold is 1. This indicates that all peptides identified for the missed protein have probabilities less than 1. Meanwhile, ProteinProphet can only identify 27 of the 31 truly present proteins. Figure 3(b) shows that the number of false positive proteins output by the iterative method decreases with the increase of high-confidence peptide selection threshold. However, ProteinProphet outputs the same number of false positive proteins regardless of the threshold. The reason is that higher selection thresholds reject more absent peptides to be included in the iteration process, and thus less absent proteins will be inferred by the iterative method. On the other hand, some false positive proteins are removed with the elimination-rule in the iteration process, while they are retained by ProteinProphet. The elimination-rule will be introduced later.

**Figure 4:**
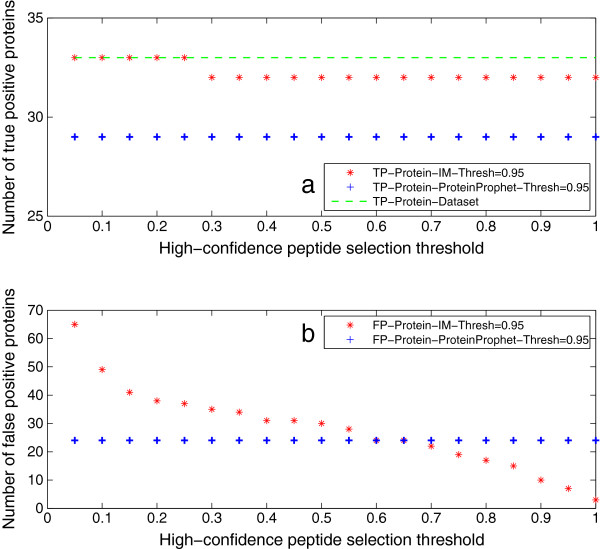
**Protein inference results of Mix 2.** This figure shows the protein inference results of Mix 2. The protein identification threshold is set as 0*.*95. They demonstrate the same patterns as the results of Mix 1. Based on the protein inference results of the two datasets, IM shows the potential of better performance than ProteinProphet in terms of the number of true and false positive proteins.

**Figure 5:**
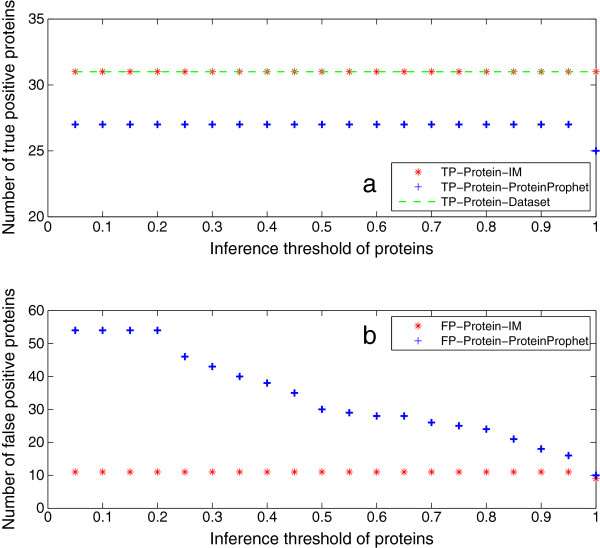
**Protein inference results of Mix 1 by varying protein inference threshold.** This figure shows the protein inference results of Mix 1 when the protein inference threshold is varied. The threshold for selecting high-confidence peptides is set as 0*.*95. It shows that the iterative method consistently outputs more true positive proteins and less false positive proteins than ProteinProphet.

**Figure 6:**
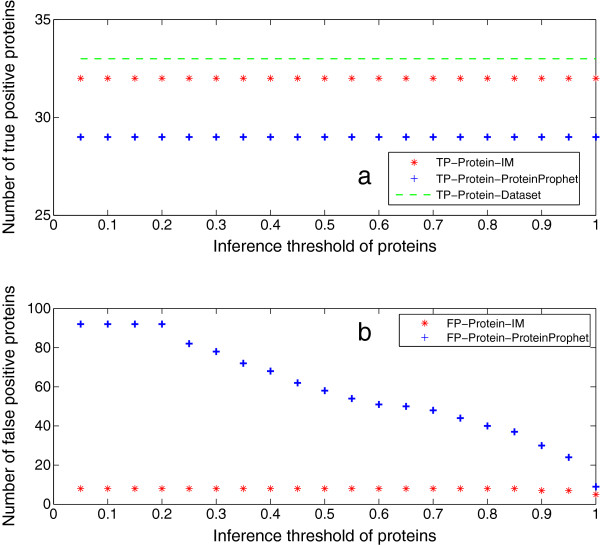
**Protein inference results of Mix 2 by varying protein inference threshold.** This figure shows the protein inference results of Mix 2 when the protein inference threshold is varied. The threshold for selecting high-confidence peptides is set as 0*.*95. They demonstrate the same patterns as the results of Mix 1.

Protein inference results of Mix 2 are shown in Figure 4. They demonstrate the same patterns as the results of Mix 1. Based on the protein inference results of the two datasets, the iterative method shows better performance than ProteinProphet in terms of the number of true and false positive proteins. In addition, we also show the number of true positive and false positive inferred proteins by varying the protein inference threshold, which are given in Figure 5 and 6. Here, the threshold for selecting high-confidence peptides is set as 0*.*95. It shows that the iterative method consistently outputs more true positive proteins and less false positive proteins than ProteinProphet as well.

### Peptide identification results

**Figure 7:**
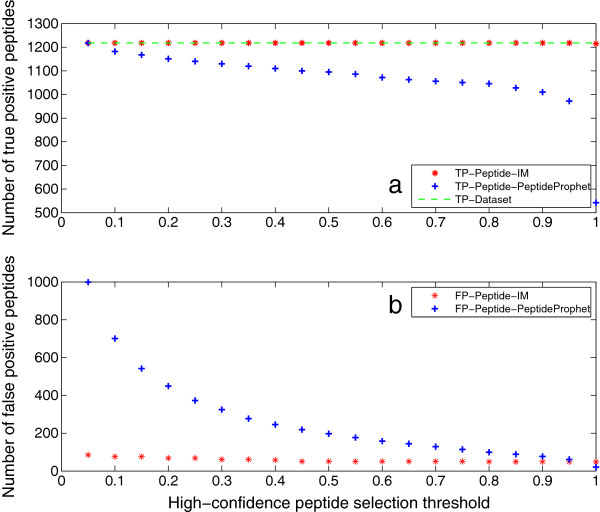
**Peptide identification results of Mix 1.** This figure illustrates the peptide identification results of Mix 1. Figure 5**(a)** shows that IM outputs all true peptides at any threshold but 1, while the number of true peptides output by PeptideProphet deceases with the increase of threshold. The reason is that IM recomputes the probability of false negative peptides by using extra information from the identified proteins. The fact that it cannot output all true peptides at the threshold of 1 agrees with that one true protein is missed at this threshold. Figure 5**(b)** shows that IM can produce much less false positives than PeptideProphet at all thresholds but 1. At threshold 1, although IM outputs 8 false positive proteins (see Figure 3(b)), less than 16 from ProteinProphet, its generation of more false positive peptides is due to the feedback framework which unifies the peptide and protein identification results. That is, negative peptides from false positive proteins are output as false positives, while for PeptideProphet, peptides are selected only by their probabilities. The feedback framework also explains why the number of false positives from IM is steady *versus* threshold, that is because these peptides are from the few counterpart proteins.

Peptide identification results are given in this section. Figure 7(a) shows that the iterative method outputs all true peptides at any threshold but 1, while the number of true peptides output by PeptideProphet deceases with the increase of threshold. The reason is that the iterative method recomputes the probability of false negative peptides by using extra information from the identified proteins. The fact that it cannot output all true peptides at the threshold of 1 agrees with that one truly present protein is missed at this threshold. Figure 7(b) shows that the iterative method can produce much less false positives than PeptideProphet at all thresholds but 1. At the threshold of 1, the iterative method generates more false positive peptides than PeptideProphet. However, it is shown in Figure 3(b) that it outputs less false positive proteins than ProteinProphet (8 *versus* 16). The reason behind this is that the feedback framework always unifies the identified peptides with the inferred proteins. More specifically, there are more false positive peptides mapped to the 8 false positive proteins from the iterative method than those mapped to the 16 ones from ProteinProphet. In other words, negative peptides from false positive proteins are output as false positives in the iterative method, while for PeptideProphet, peptides are selected only by their probabilities. The feedback framework also explains why the number of false positives from the iterative method is steady *versus* threshold, that is because these peptides are from the few counterpart proteins.

**Figure 8:**
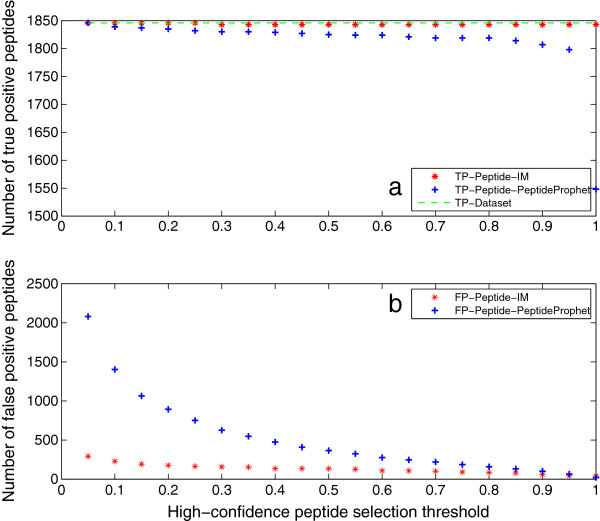
**Peptide identification results of Mix 2.** This figure illustrates the peptide identification results of Mix 2. They have the same trend as the results of Mix 1. Both peptide identification results indicate that IM can identify more true positive and less false positive peptides than PeptideProphet. This can be attributed to the feedback framework, which recomputes the probability of true peptides with low PeptideProphet probability, and eliminates negative peptides of unidentified proteins.

Peptide identification results of Mix 2 are illustrated in Figure 8. They have the same trend as the results of Mix 1. Both peptide identification results indicate that the iterative method can identify more true positive and less false positive peptides than PeptideProphet. This can be attributed to the feedback framework, which recomputes the probability of true peptides with low PeptideProphet probability, and eliminates negative peptides of unidentified proteins.

### Shared peptides

**Figure 9:**
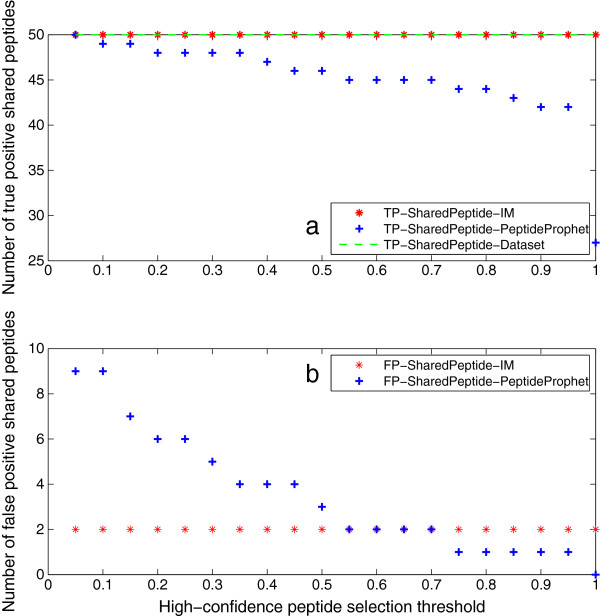
**Results of shared peptides of Mix 1.** This figure illustrates the identification results of shared peptides of Mix 1. It is shown that IM outputs all true shared peptides of Mix 1 regardless of threshold, while this number from PeptideProphet decreases rapidly with the increase of the threshold. In addition, IM outputs a constant number of false positive shared peptides. This is because these peptides are from one false positive protein, of which the constituent peptides are given in Table 2.

**Figure 10:**
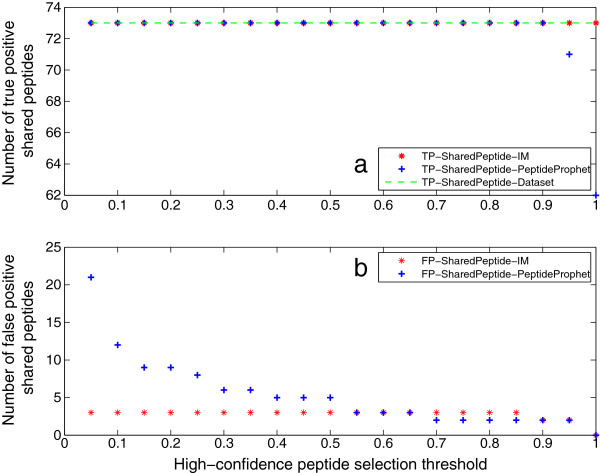
**Results of shared peptides of Mix 2.** This figure illustrates the identification results of shared peptides of Mix 2. They have a similar pattern as the results of Mix 1, except that IM can also output zero false positive as PeptideProphet. Generally, this iterative method can identify much more true shared peptides than PeptideProphet, and output very few false positives.

The identification results of shared peptides are shown in Figures 9 and 10. It is shown in Figure 9 that the iterative method outputs all true shared peptides of Mix 1 regardless of threshold, while this number from PeptideProphet decreases rapidly with the increase of the threshold. In addition, the iterative method outputs a constant number of false positive shared peptides. This is because these peptides are from one false positive protein, of which the constituent peptides are given in Table 2. This protein is false positive according to the data source [20], while it is inferred with probability 1 by ProteinProphet. The identification results of Mix 2 are given in Figure 10. They have a similar pattern as the results of Mix 1, except that the number of false positives identified by the iterative method also decreases with the increase of the threshold as PeptideProphet. Generally, this iterative method can identify much more true shared peptides than PeptideProphet, and output very few false positives. 

**Table 2:** Protein SW:K2C1_HUMAN and its constituent peptides

**Protein**	**Constituent peptides**
	SLVNLGGSK
	THNLEPYFESFINNLR
	YEELQITAGRHGDSVR
	QNEDIAQK
	TNAENEFVTIKK
SW:K2C1	**YEELQITAGR**
_HUMAN	**NKYEDEINKR**
	WELLQQVDTSTR
	SLDLDSIIAEVK
	SLNNQFASFIDK
	NKLNDLEDALQQAKEDLAR
	NKLNDLEDALQQAK
	SKAEAESLYQSK

Protein SW:K2C1_HUMAN is a false protein in the sample. This table shows the two false positive peptides (in bold) contained in the protein and its other constituent peptides identified by the iterative method.

### Convergence of the iterative method

It is not attempted here to give a mathematical proof of the convergence of the iterative method. Instead, an explanation is provided. The stop criterion is the convergence of the probabilities of the putative proteins. According to the flowchart in Figure 1, the stop criterion naturally fails if there is any change to the putative protein list. In addition, the protein inference model can assure that the probabilities of proteins with high-confidence identified peptides will converge to 1. Therefore, the convergence of the protein probabilities is reduced to reaching the steady state of the protein list. Since the protein list is produced by using a group of peptides to find their parent proteins, this stop criterion can be further reduced to reaching the steady state of the peptide list.

The steady state of the peptide list is guaranteed. Peptides can be classified into unique peptides and shared peptides. First, we will see that shared peptides can remain steady in the list. There are three kinds of shared peptides in the iteration process: shared by both negative and positive, by negative and by positive proteins. (Given the threshold of high-confidence proteins, proteins with probability equal to or greater than the threshold are classified as positive; otherwise, negative). If a peptide is only shared by negative proteins, then it will not be selected into the high-confidence peptide list; if a peptide is only shared by positive proteins, then it will be selected and remains steady in the peptide list. If a peptide is shared by both negative and positive proteins, then it will be included and eventually remains steady in the peptide list. This is assured by the discovery and application of an elimination-rule for negative proteins which share peptides with positive proteins. During the iteration, some shared peptides are selected into the process by positive proteins. Then, these peptides are used to search proteins, and negative proteins will be introduced into the iteration process. After several iterations, these negative proteins will be removed because of their low probabilities produced by the protein inference model. However, they will be re-selected into the cycle due to the shared peptides from positive proteins. Therefore, if these proteins are allowed to enter the iteration process, they will always be “in and out” of the putative protein list. Proteins with such pattern will be eliminated from the iteration process. This elimination-rule can rule out negative proteins, which are usually also absent proteins, and thus increase the chance of assigning shared peptides to truly present proteins. After the removal of those false proteins, shared peptides will only be considered from positive proteins, and thus shared peptides can remain steady in the peptide list.

Similarly, there are three kinds of unique peptides: unique to positive proteins, unique to negative proteins with no peptides shared with positive proteins and unique to negative proteins with shared peptides from positive proteins. In these three situations, only unique peptides from positive proteins will stay steady in the high-confidence peptide list. Unique peptides from negative proteins with no shared peptides from positive proteins will not be selected at all; while if the negative proteins with shared peptides from positive proteins, these unique peptides will be eliminated eventually with the removal of these proteins by the elimination-rule. Therefore, unique peptides can also remain steady in the peptide list, and this completes the explanation of the convergence of the iterative method.

In addition, we briefly account the scalability of our method here. Before beginning the iterative method, we need to construct two hash tables which are used to organize the information about each peptide and each protein from the Sequest reports and PeptideProphet probabilities, the complexity of which are *O*(*MN*) and *O*(*N*), respectively. When running the iterative method, the cost of each iteration is (*O*(*M*×*max*(*M*_*i*_)) + *O*(*N*×*max*(*n*_*k*_)), *i*=1…*M*;*k*=1…*N*), where *M* and *N* are the total number of peptides and proteins involved in the iteration process, respectively; and *M*_*i*_ is the number of parent proteins of peptide P_*i*_, and *n*_*k*_ is the number of peptides mapped to protein Q_*k*_.

## Conclusion

This paper proposed a unified feedback framework for protein inference based on peptides identified from tandem mass spectra, and an iterative method is implemented to process Sequest peptide identification reports according to this framework. This method outputs a list of peptides and a list of counterpart proteins simultaneously. Based on the two datasets from standard proteins, the results have shown that the iterative method performs superiorly to the popular programs PeptideProphet and ProteinProphet in identifying peptides and proteins. However, at this point, the implementation of the iterative method is not ready for the practical use on identifying peptides and proteins like PeptideProphet and ProteinProphet. First, it is not tested with complex datasets yet, so the rigor of this method needs more examination. Secondly, it is mainly developed for testing the framework, not for direct-use like those programs. Based on the results we got, there is obvious advancement of this method, and we will leave the development of a practical implementation as the future work.

## Competing interests

The authors declare that they have no competing interests.

## Author’s contributions

JS developed the algorithm, designed and executed all experimental work, and wrote the first draft. FXW supervised and initiated the project, and revised the manuscript. Both authors read and approved the manuscript.
